# Targeting Fibroblast Growth Factor 23 Signaling with Antibodies and Inhibitors, Is There a Rationale?

**DOI:** 10.3389/fendo.2018.00048

**Published:** 2018-02-20

**Authors:** Seiji Fukumoto

**Affiliations:** ^1^Department of Molecular Endocrinology, Fujii Memorial Institute of Medical Sciences, Institute of Advanced Medical Sciences, Tokushima University, Tokushima, Japan

**Keywords:** hypophosphatemia, rickets, osteomalacia, antibody, chronic kidney disease-mineral and bone disorder (CKD-MBD)

## Abstract

Fibroblast growth factor 23 (FGF23) is a phosphotropic hormone mainly produced by bone. FGF23 reduces serum phosphate by suppressing intestinal phosphate absorption through reducing 1,25-dihydroxyvitamin D and proximal tubular phosphate reabsorption. Excessive actions of FG23 result in several kinds of hypophosphatemic rickets/osteomalacia including X-linked hypophosphatemic rickets (XLH) and tumor-induced osteomalacia. While neutral phosphate and active vitamin D are standard therapies for child patients with XLH, these medications have several limitations both in their effects and adverse events. Several approaches that inhibit FGF23 actions including anti-FGF23 antibodies and inhibitors of FGF signaling have been shown to improve phenotypes of model mice for FG23-related hypophosphatemic diseases. In addition, clinical trials indicated that a humanized anti-FGF23 antibody increased serum phosphate and improved quality of life in patients with XLH. Furthermore, circulatory FGF23 is high in patients with chronic kidney disease (CKD). Many epidemiological studies indicated the association between high FGF23 levels and various adverse events especially in patients with CKD. However, it is not known whether the inhibition of FGF23 activities in patients with CKD is beneficial for these patients. In this review, recent findings concerning the modulation of FGF23 activities are discussed.

## Introduction

Fibroblast growth factor 23 (*FGF23*) was identified as a responsible gene for autosomal dominant hypophosphatemic rickets (ADHR) in 2000 by positional cloning (1). FGF23 was also cloned as a responsible humoral factor for tumor-induced osteomalacia (TIO), a rare paraneoplastic syndrome (2). ADHR and TIO are diseases characterized by hypophosphatemia associated with impaired renal tubular phosphate reabsorption. Subsequent studies established that FGF23 is a hormone mainly produced by bone and regulates phosphate and vitamin D metabolism by binding to Klotho–FGF receptor complex (3–7). Klotho is a single membrane spanning protein. While there is also soluble Klotho, its role in FGF23 signaling is not clear (8). FGF23 was shown to transduce signals only in tissues which express membrane form Klotho (6). FGF23 reduces serum phosphate by inhibiting intestinal phosphate absorption through decreasing serum 1,25-dihydroxyvitamin D [1,25(OH)_2_D] level and proximal tubular phosphate reabsorption (3). Therefore, ADHR and TIO have been considered to be caused by excessive actions of FGF23. These results lead to develop a concept that the inhibition of FGF23 activities may be beneficial for hypophosphatemic patients caused by excessive actions of FGF23. Furthermore, it has been shown that FGF23 is high in patients with chronic kidney disease (CKD) and can be extremely high in some subjects with end-stage renal disease (ESRD) (9, 10). Circulatory FGF23 levels have been shown to be associated with various adverse events such as higher mortality, cardiovascular events, and left ventricular hypertrophy, especially in subjects with CKD. However, it is not known whether these events are direct consequences of FGF23 activities (11). In this review, the significance of inhibiting FGF23 activities in various diseases is discussed.

## FGF23-Related Hypophosphatemic Diseases

Chronic hypophosphatemia is an important cause for rickets and osteomalacia characterized by impaired mineralization of bone matrix. Rickets develops in children before the closure of epiphyseal plate and results in growth retardation and bone deformities. In contrast, osteomalacia in adults can cause severe muscle weakness and bone pain. Serum phosphate level is maintained by intestinal phosphate absorption, renal tubular phosphate handling and equilibrium between extracellular phosphate and phosphate in bone or intracellular fluid. Of these, renal phosphate handling is the main determinant of chronic serum phosphate level. Most phosphate filtered through glomeruli is reabsorbed in proximal tubules by type 2a and 2c sodium-phosphate cotransporters. FGF23 reduces the expression of these sodium-phosphate cotransporters and suppresses phosphate reabsorption (3). At the same time, FGF23 reduces serum 1,25(OH)_2_D by modulating the expression of vitamin D-metabolizing enzymes (3). FGF23 suppresses the expression of *CYP27B1* that encodes a protein responsible for the production of 1,25(OH)_2_D. FGF23 also enhances *CYP24A1* expression that encodes an enzyme that works to reduce 1,25(OH)_2_D level.

After the identification of FGF23, several kinds of enzyme-linked immunosorbent assay for FGF23 have been established (12, 13). A part of FGF23 protein is proteolytically cleaved into inactive N-terminal and C-terminal fragments before or during the process of secretion. FGF23 level can be regulated by both *FGF23* transcription and this posttranslational processing of FGF23 protein. For example, iron deficiency seems to enhance *FGF23* production and also the processing of FGF23 protein (14). Therefore, FGF23 level does not always reflect the amount of *FGF23* transcription. Intact assay using two kinds of antibodies that recognize N-terminal and C-terminal portions of the processing site of FGF23 detects only full-length biologically active FGF23 (12). In contrast, C-terminal assay using antibodies against the C-terminal part of FGF23 measures both full-length and processed inactive C-terminal fragment of FGF23 (13). FGF23 level measured by C-terminal assay seems to correlate with the amount of *FGF23* transcription. Intravenous iron preparations inhibit gene expression of *FGF23*. While iron dextrane does not affect the cleavage of FGF23, several iron preparations such as iron polymaltose and iron carboxymaltose may block the cleavage, resulting in paradoxical hypophosphatemia (14). This two step regulation of FGF23 needs to be kept in mind when interpreting results of FGF23 levels by intact and C-terminal FGF23 assays. Measurement of FGF23 levels in patients with chronic hypophosphatemia indicated that FGF23 levels are high in hypophosphatemic patients with ADHR and TIO by both assays (12, 13, 15). In contrast, FGF23 levels are low in patients with chronic hypophosphatemia from other causes such as vitamin D deficiency and Fanconi syndrome indicating that circulatory FGF23 level is suppressed in these patients by hypophosphatemia or other accompanying metabolic changes (16). From these results, high FGF23 in patients with chronic hypophosphatemia seemed to indicate that this hypophosphatemia is caused by excessive actions of FGF23.

In addition to patients with ADHR and TIO, FGF23 levels have been shown to be high in several kinds of hypophosphatemic diseases (Table 1). Of these, X-linked hypophosphatemic rickets (XLH) caused by inactivating mutations of *phosphate-regulating gene with homologies to endopeptidases on the X chromosome (PHEX)* is the most prevalent cause of genetic hypophosphatemic disease. More than three hundred kinds of mutations in *PHEX* have been assembled in a database.^1^ PHEX is a single membrane spanning protein mainly expressed in bone and teeth (17). There is a murine model of XLH called *Hyp*. *Hyp* mice show similar biochemical features to those of patients with XLH. Genetic analysis indicated that there is a deletion in 3′ region of *Phex* gene in *Hyp* mice (18). It has been shown that *Fgf23* is overexpressed in bone and circulatory Fgf23 is high in *Hyp* mice (19). Therefore, it is believed that inactivating mutations in *PHEX* somehow induce enhanced expression of *FGF23* in bone and cause excessive actions of FGF23 in patients with XLH. Signals from FGF receptor was reported to be involved in the overproduction of FGF23 production in *Hyp* mice (7). However, the precise detailed role of PHEX in the regulation of *FGF23* expression needs to be established.

**Table 1:** FGF23-related hypophosphatemic diseases.

	Responsible gene	Mutations	Reference
X-linked hypophosphatemic rickets: XLH	*PHEX*	Inactivating	(20)
Autosomal dominant hypophosphatemic rickets: ADHR	*FGF23*	Activating	(1)
Autosomal recessive hypophosphatemic rickets 1: ARHR1	*DMP1*	Inactivating	(21, 22)
Autosomal recessive hypophosphatemic rickets 2: ARHR2	*ENPP1*	Inactivating	(23, 24)
Osteoglophonic dysplasia	*FGFR1*	Activating	(25)
Jansen-type metaphyseal chondrodysplasia	*PTH1R*	Activating	(26)
Hypophosphatemia with dental abnormality and ectopic calcification	*FAM20C*	Inactivating	(27)
McCune-Albright syndrome	*GNAS1*	Activating	(28)
Epidermal nevus syndrome: ENS	*HRAS, KRAS, NRAS*	Activating	(29)
Tumor-induced osteomalacia: TIO	*FN-FGF1, FN-FGFR1*	Activating	(30)
Hypophosphatemia after infusion of saccharated ferric oxide, iron polymaltose, or ferric carboxymaltose			
Biliary atresia			

*PHEX, phosphate-regulating gene with homologies to endopeptidases on the X chromosome; FGF23, fibroblast growth factor 23; DMP1, dentin matrix protein 1; ENPP1, ectonucleotide pyrophosphatase/phosphodiesterase 1; FGFR1, fibroblast growth factor receptor 1; PTH1R, parathyroid hormone 1 receptor; FAM20C, family with sequence similarity 20, member C; GNAS1, guanine nucleotide binding protein, alpha-stimulating activity polypeptide 1; HRAS, Harvey rat sarcoma viral oncogene homolog; KRAS, Kirsten rat sarcoma rival oncogene homolog: NRAS, neuroblastoma RAS viral oncogene homolog; FN, fibronectin; FGFR, FGF receptor*.

Mutations in *dentin matrix protein 1 (DMP1)* and *ectonucleotide pyrophosphatase/phosphodiesterase 1 (ENPP1)* results in autosomal recessive hypophosphatemic rickets 1 and 2, respectively (21–24). Furthermore, mutations in several other genes have been shown to cause hypophosphatemic diseases with high FGF23 levels (31). Inactivating mutations in *FAM20C* was reported in Raine syndrome, a usually lethal osteosclerotic disease (32). However, hypophosphatemia with high FGF23 was later reported in some surviving patients (27). Osteoglophonic dysplasia is caused activating mutations in *FGF receptor 1*. Hypophosphatemia with high FGF23 in this disease again suggests the involvement of signals from FGF receptor in FGF23 production (25). Epidermal nevus syndrome is caused by somatic mutations in *RAS* oncogenes and is characterized by sebaceous nevi and skeletal defects (29). These oncogene products can transduce signals from receptor tyrosine kinases including FGF receptor. Jansen-type metaphyseal chodrodysplasia and McCune-Albright syndrome are caused by activating mutations in *parathyroid hormone* (*PTH*) *1 receptor* and *GNAS1*, respectively (26, 28). These results suggest that cyclic AMP pathway is involved in FGF23 production. In some patients with TIO, *FN (fibronectin)-FGF receptor 1* or *FN-FGF1* fusion gene was reported in responsible tumors (30). It is likely that these genes activate some intracellular signaling pathway to enhance FGF23 production.

In addition to diseases with known genetic causes, hypophosphatemia with high FGF23 has been reported in patients receiving some intravenous iron preparations (33, 34). Recently, it has been reported that biliary atresia can be associated with hypophosphatemia with high FGF23 (35). In most of these FGF23-related hypophosphatemic diseases, FGF23 is considered to be overexpressed in bone while the detailed mechanism of this overproduction is not clear. On the contrary, in patients with TIO, the responsible tumors produce FGF23 and FGF23 is shown to be expressed in liver in a patient with biliary atresia. Collectively, these results indicate that excessive production and actions of FGF23 can cause several kinds of hypophosphatemic diseases.

## The Inhibition of FGF23 Activity as a New Therapeutic Maneuver for FGF23-Related Hypophosphatemic Diseases

### Direct FGF23 Targeting

Tumor-induced osteomalacia is a paraneoplastic syndrome and can be cured by complete resection of the responsible tumors. However, it is sometimes difficult to find the responsible tumors in patients with TIO. Even when the responsible tumors can be found, it is not always possible to completely remove the lesions. For those patients with TIO whose responsible tumors cannot be removed, neutral phosphate and active vitamin D are usually prescribed. For patients with most other FGF23-related hypophosphatemic diseases including XLH, the same drugs are also used. However, these medications are not drugs based on the pathophysiology of these diseases. In addition, these medications may not able to correct impaired growth completely in patients with hypophosphatemic rickets (36). Furthermore, administration of phosphate and active vitamin D can be associated with several adverse events such as hypercalcemia, hypercalciuria, nephrocalcinosis, and gastrointestinal symptoms (37).

It has been shown that excessive activities of FGF23 underlie the pathogenesis of FGF23-related hypophosphatemic diseases as mentioned above. Therefore, the suppression of FGF23 activities has been considered as a novel therapy for patients with these diseases. Murine monoclonal antibodies against N-terminal and C-terminal portions of human FGF23 were obtained. These antibodies inhibited *in vitro* FGF23 activity and synergistically increased serum phosphate and 1,25(OH)_2_D levels in normal mice (38). These results as well as phenotypes of hyperphosphatemia and high 1,25(OH)_2_D in *Fgf23* null mice confirmed that FGF23 is a physiological regulator of phosphate and vitamin D metabolism (4, 38). Then, these antibodies were tested in *Hyp* mice and were shown to increase serum phosphate and 1,25(OH)_2_D (39). In addition, repeated administration of these antibodies enhanced longitudinal growth of long bones, increased bone mineral density and corrected impaired mineralization of *Hyp* mice (39). Furthermore, these antibodies increased grip power and spontaneous movement of *Hyp* mice (40). These results suggested that the inhibition of FGF23 activities by FGF23 antibodies can ameliorate biochemical, morphological, histological and clinical abnormalities of patients with FGF23-related hypophosphatemic rickets/osteomalacia.

Based on the results of these preclinical studies, humanized anti-FGF23 monoclonal antibody (burosumab) was developed and tested in several clinical trials. The initial phase I study involving 38 adult patients with XLH showed that burosumab increased serum phosphate, 1,25(OH)_2_D and tubular maximum transport of phosphate per glomerular filtration rate (TmP/GFR), an index of proximal tubular phosphate reabsorption, in a dose-dependent manner (41). Subsequent study with 28 adult patients with XLH showed that subcutaneous injections of burosumab every 4 weeks increased serum phosphate, 1,25(OH)_2_D and TmP/GFR after each administration (42). Furthermore, burosumab was shown to significantly improve patient perception of physical functioning and stiffness (43). These results indicated that burosumab can improve biochemical abnormalities of adult patients with XLH and improve quality of life. However, it was unclear from these studies whether burosumab can also work in children and ameliorate rickets/osteomalacia.

While the results have not yet been published as articles so far, several clinical trials with burosumab are ongoing. Results presented in several scientific meetings and available on the web suggest that burosumab can improve biochemical abnormalities in child patients with XLH and also in patients with TIO.^2^,^3^ In addition, burosumab seems to improve roentgenological signs of rickets in children. Based on these results, the US Food and Drug Administration granted a Breakthrough Therapy Designation for burosumab in 2016 (see footnote 2). However, there are several questions to be answered in the future studies. Long-term safety should be carefully monitored. In addition, it has not yet been shown whether burosumab can heal rickets/osteomalacia and normalize height in child patients with XLH. Furthermore, it has not been established how long burosumab should be used for child patients with XLH. Theoretically, burosumab seems to be effective in patients with other FGF23-related hypophosphatemic diseases than XLH and TIO. This also needs to be proved in the future trials. Collectively, burosumab has been shown to inhibit FGF23 actions in human and seems to be promising as a novel therapy for patients with FGF23-related hypophosphatemic diseases.

In addition to anti-FGF23 antibody, a computationally identified compound binding to FGF23 was also shown to increase serum phosphate and 1,25(OH)_2_D in a model mouse of FGF23-related hypophosphatemic diseases (44). These compounds can be cost-effective compared to the antibody. However, this small molecule has not been used in clinical trials.

### Klotho–FGF Receptor Complex Targeting

Another approach is to inhibit the binding of FGF23 to Klotho–FGF receptor complex. Purified C-terminal fragment of FGF23 protein was shown to compete with full-length FGF23 for the binding to Klotho–FGF receptor complex (45). Administration of this C-terminal fragment of FGF23 temporally increased serum phosphate of *Hyp* mice (45). The C-terminal portion of FGF23 protein was fused to Fc portion of IgG1 to increase the stability of the C-terminal fragment (46). This FGF23 c-tail-Fc fusion molecule also increased serum phosphate and improved mineralization of *Hyp* mice when repeatedly injected (46). In addition to this C-terminal fragment of FGF23, several small compounds were shown to impair the interaction between FGF23 and Klotho–FGF receptor complex (47).

### Targeting FGF Receptor

In addition, the inhibition of FGF receptor was shown to inhibit FGF23 activities (48, 49). Several reports suggest that signals from FGF receptor stimulate FGF23 production in bone (7, 48). Therefore, it is possible that the inhibition of FGF receptor suppresses both the production and the actions of FGF23. NVP-BGJ398, a pan FGF receptor inhibitor, increased serum phosphate, enhanced bone growth, increased mineralization, and corrected the disturbed growth plate structure in *Hyp* mice (49).

### Targeting of Downstream Signals from FGF Receptor

The inhibition of mitogen-activated protein kinase (MAPK) pathway was also shown to impair FGF23 actions while FGF receptor can activate several signal transduction pathways. PD0325901, an inhibitor of MAPK pathway, was shown to increase serum phosphate and 1,25(OH)_2_D, and correct impaired mineralization in *Hyp* mice (50). These preclinical studies indicated that there are several ways to inhibit excessive FGF23 activities causing FGF23-related hypophosphatemic diseases.

However, FGF receptor-MAPK pathway is involved in numerous biological processes in addition to mediating FGF23 actions suggesting that the inhibition of this pathway can be associated with various adverse events. NVP-BGJ398 and PD0325901 have been tested in several clinical trials mainly for patients with solid cancers.^4^ NVP-BGJ398 was shown to inhibit FGF23 secretion in patients with TIO caused by malignant tumors (51). However, these inhibitors have not yet tested for patients with genetic FGF23-related hypophosphatemic diseases or TIO caused by benign tumors. FGF23 c-tail-Fc fusion molecule and other small compounds that impair the binding of FGF23 to Klotho–FGF receptor complex have not been tested in clinical trials, either.

## FGF23 and CKD-MBD

Circulatory FGF23 level is high in patients with CKD. Several studies indicated that FGF23 starts to increase in the early phase of CKD (10, 52). This increase of FGF23 precedes those of PTH and phosphate. While the detailed regulatory mechanisms of FGF23 production and circulatory FGF23 levels remain to be clarified, 1,25(OH)_2_D and high phosphate diet were shown to enhance FGF23 levels (53). However, in patients with early CKD with high FGF23 level, 1,25(OH)_2_D and phosphate are not high. Recent studies suggest that inflammation and iron deficiency are involved in this high FGF23 in patients with CKD (54).

In a model rat of early CKD, the inhibition of Fgf23 activity by anti-FGF23 antibodies were shown to enhance proximal tubular phosphate reabsorption and increase serum phosphate and 1,25(OH)_2_D (55). These results suggest that high FGF23 in patients with early CKD suppresses phosphate reabsorption and works to prevent the development of hyperphosphatemia. The inhibition of Fgf23 activities by anti-FGF23 antibody in a model rat of CKD was also shown to cause higher mortality by increasing serum phosphate and promoting ectopic calcification (56). Therefore, this increase of FGF23 in patients with early CKD is considered to be one of adaptive responses to maintain mineral homeostasis.

In contrast, there are now many epidemiological studies that indicate the association between high FGF23 levels and various adverse events especially in patients with CKD or ESRD (11). For example, high FGF23 levels in patients who were beginning hemodialysis were shown to be associated with higher mortality during the first year of hemodialysis (57). Adverse events associated with high FGF23 are quite diverse including cardiovascular events, left ventricular hypertrophy, progression of CKD, fractures, higher mortality, frailty, insulin resistance, and so on. These associations suggest that the inhibition of FGF23 activity may be beneficial in patients with CKD under certain conditions. Actually, secondary analysis of Evaluation Of Cinacalcet Hydrochloride Therapy to Lower CardioVascular Events indicated that cinacalcet-induced reductions in serum FGF23 were associated with lower rates of cardiovascular death and major cardiovascular events (58). However, there has been no study that examined effects of inhibiting FGF23 activities by anti-FGF23 antibody or inhibitors in patients with ESRD. In addition, the reason for the association between high FGF23 levels and various adverse events are not clear enough. While it was reported that FGF23 signals in a Klotho-independent fashion through FGFR4/nuclear factor of activated T cells/calcineurin, which may cause left ventricular hypertrophy (59, 60), it has not been shown how FGF23 can activate FGF receptor without Klotho. In addition, it is not clear either whether FGF23 induces various adverse events directly working on several tissues like heart, bone and kidney. Further studies are necessary to establish the usefulness of inhibiting FGF23 activities in patients with CKD.

## Conclusion

Fibroblast growth factor 23 is a hormone that reduces serum phosphate and 1,25(OH)_2_D levels. For patients with excessive hormone actions, therapeutic measures to suppress the activities of that hormone are used. Therefore, it is reasonable to develop methods to inhibit FGF23 actions for patients with FGF23-related hypophosphatemic diseases. Patients with hypophosphatemic disorders by FGF23 excess may benefit from FGF23 blocking antibodies or inhibitors of FGF23 signaling. However, these therapies need careful monitoring because deficient actions of FGF23 result in hyperphosphatemic disease. On the contrary, FGF23 seems to be high in response to changes of mineral homeostasis or other metabolic process in patients with CKD. Patients with CKD may benefit from these novel therapies to the extent that FGF23 secretion is excessively stimulated and beyond what’s needed in compensation. However, there is not enough evidence that indicates the inhibition of FGF23 activities is useful for patients with CKD.

## Author Contributions

The author confirms being the sole contributor of this work and approved it for publication.

## Conflict of Interest Statement

The department is supported by Chugai Pharmaceutical Co., Ltd., Taisho Pharmaceutical Co., Ltd., Ono Pharmaceutical Co., Ltd., and Kyowa Hakko Kirin Co., Ltd.

## Footnotes

^1^http://www.phexdb.mcgill.ca/.

^2^http://www.ultragenyx.com/pipeline/krn23-xlh/.

^3^http://www.ultragenyx.com/pipeline/krn23-tio/.

^4^https://clinicaltrials.gov/ct2/home.
